# A Large Mullerian Cyst With Pressure Symptoms: A Case Report

**DOI:** 10.7759/cureus.32917

**Published:** 2022-12-25

**Authors:** Indira Prasad, Smita Singh, Shivangi Sinha, Tarun Kumar, Ishita Roy

**Affiliations:** 1 Obstetrics and Gynaecology, All India Institute of Medical Sciences, Patna, IND; 2 Pathology/Lab Medicine, All India Institute of Medical Sciences, Patna, IND

**Keywords:** gartner’s cyst, vaginal cyst, dysuria, pressure symptoms, mullerian cyst

## Abstract

Mullerian cysts are of embryological origin and are usually found incidentally during delivery or a routine gynecological examination. They remain asymptomatic unless they become large enough to cause heaviness or pressure on the surrounding structures. Here, we present the case of a large 8 × 5 cm vaginal cyst that presented with bladder and bowel pressure symptoms. Complete vaginal cyst excision through the vaginal route was done to prevent its recurrence. The histopathology report showed ciliated columnar mucinous epithelium and confirmed the vaginal cyst as a cyst of Mullerian origin.

## Introduction

Vaginal cysts are rare with a reported prevalence of one in 200 [1]. Because it mostly remains asymptomatic as a small single cyst (77.5%) [2], the actual prevalence may be underestimated. However, larger cysts (>4 cm) may cause heaviness in the perineal region, discomfort, a feeling of swelling at the vulva, dyspareunia, voiding difficulties, or vaginal discharge, and comes to notice.

Vaginal cysts are either congenital or acquired. Based on histopathology, the congenital cyst may be of Mullerian, paramesonephric, or urothelial origin. Literature suggests that the Mullerian cyst is the most common type, accounting for one-third of all benign vaginal cysts [3]. It is mostly lined by endocervical and, occasionally, fallopian tube epithelium [4]. A Mullerian cyst develops commonly in the third or fourth decade of life.

In embryonic life, both the mesonephric duct and the paramesonephric duct remain in the female fetus. Between the eighth to the ninth week, the mesonephric duct begins to degenerate in the female in the absence of testosterone, while two paramesonephric ducts or Mullerian ducts fuse to form the uterus, cervix, upper vagina, vestibule, and the female urethra. During vaginal development, the Mullerian ducts join the urogenital sinus from where the sinovaginal bulbs evaginate and proliferate cranially to form the lower third of the vagina. Subsequently, the squamous epithelium of the urogenital sinus replaces the mucinous columnar epithelium of the Mullerian duct. Any remnant foci of Mullerian epithelium in the lower vagina may persist and form cysts over a long time due to the secretion of mucinous content. This columnar epithelial lining in any vaginal cyst wall with mucinous content is the crucial differentiating feature for cysts of Mullerian origin [5]. Sometimes it expands in size to cause dyspareunia, increased frequency, a sense of incomplete evacuation, obstructed urinary flow, and dysuria [2]. Less commonly, it may cause altered stool habits, as seen in the reported case due to the pressure effect.

Here, we are reporting a unique case of a sizeable vaginal cyst leading to voiding difficulty and increased frequency of defecation, which is a very rare presentation. It initially looked like pelvic organ prolapse, and later, after clinical examination, a diagnosis of Gartner’s duct cyst was considered due to its location in the lateral pelvic wall. However, it was finally identified as a Mullerian cyst on histopathological examination.

## Case presentation

A 43-year-old female, with a parity of two and a history of two spontaneous abortions, ligated 10 years back, visited our outpatient department with the complaint of a vaginal mass associated with obstructed urinary flow, increased urinary frequency, dysuria, burning micturition, incomplete evacuation of bladder and vaginal discharge in the last two months. She first noticed the vaginal swelling during her first pregnancy 14 years ago. At that time, it was asymptomatic and very small (pea-size), and all the deliveries were uneventful. No treatments were therefore needed. In due course, she noticed a significant increase in its size, especially over the past year. She also complained of loose stools and frequent bowel movements for the past six months and was concerned as her symptoms were not relieved, although she had consulted a few physicians for this. She had never complained of dyspareunia. The urinary problems and the frequent bowel movements had prompted her to seek consultation.

**Figure 1:**
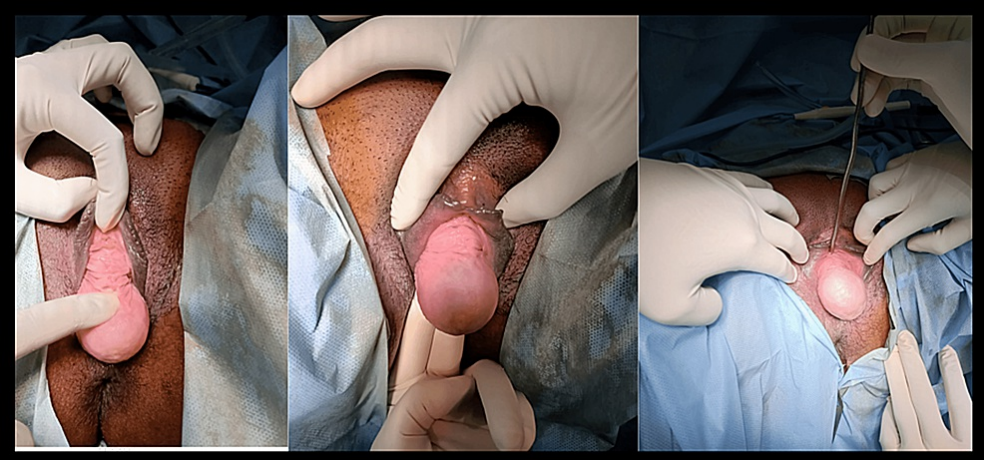
Anterior vaginal cyst 3 cm away from the urethra (left). Urethra separate from the cyst (middle). Cyst protruding 4 cm outside introitus mimicking cystocele (right).

On perineal inspection, external genitalia was normal. A smooth bulge just below the urethra was emerging about 4 cm outside the introitus. On first look, it mimicked pelvic organ prolapse. On per speculum examination, about 8 × 5 cm of cystic swelling was seen protruding from the left anterolateral wall of the vagina, 3 cm below the urethral meatus (Figure 1).

On palpation, it was cystic in consistency, sessile, and non-tender. The cervix and uterus were normal. On transperineal ultrasonography, an anechoic cyst of 8 × 5 cm distinct from the uterus, cervix, and other surrounding structures was detected. With a provisional diagnosis of a large vaginal cyst, most likely Gartner’s cyst, we planned for a complete vaginal cyst excision.

The patient’s routine blood evaluations and urine analysis were normal. Her blood group was O-negative, so one blood donor was arranged. Any associated genitourinary defects were excluded by ultrasonography. No MRI of the pelvis area was conducted as it was costly and would not change the treatment plan. A small intramural myoma of 1.5 cm was incidentally found, which was asymptomatic. Consent for bladder injury was taken because of proximity.

**Figure 2:**
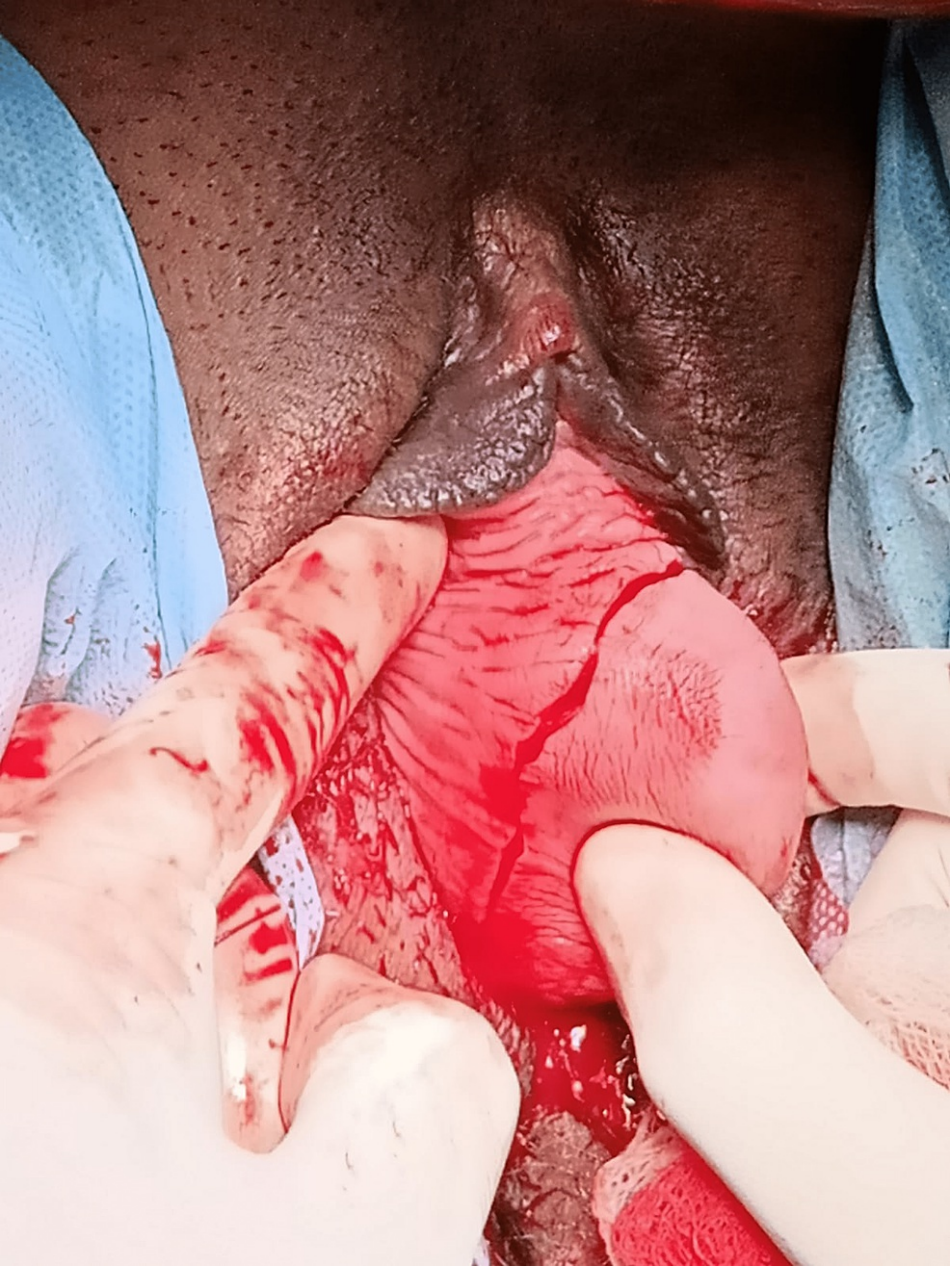
Curvilinear circumferential incisions performed.

**Figure 3:**
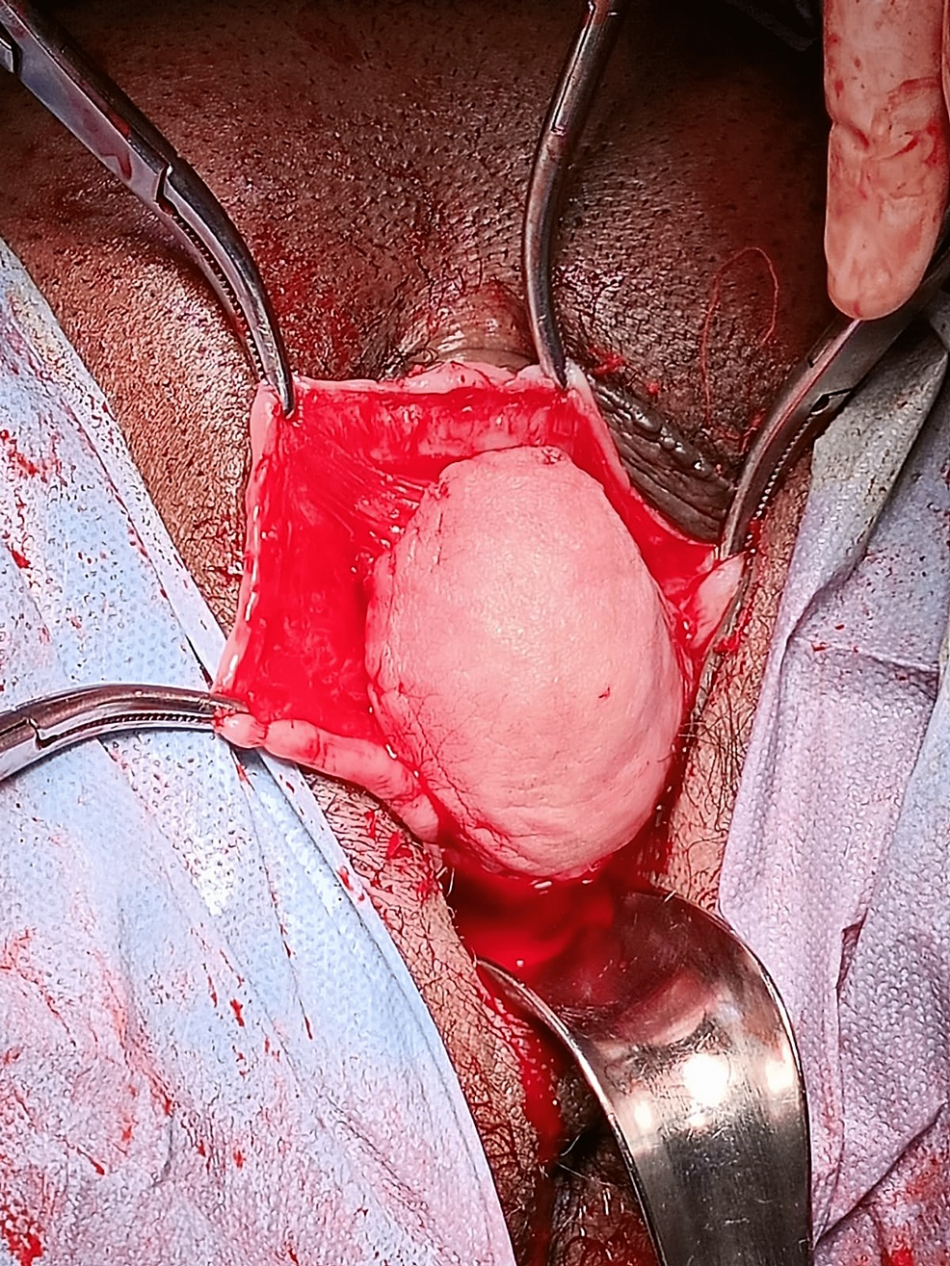
Vaginal flap separated anteriorly from the cyst wall.

**Figure 4:**
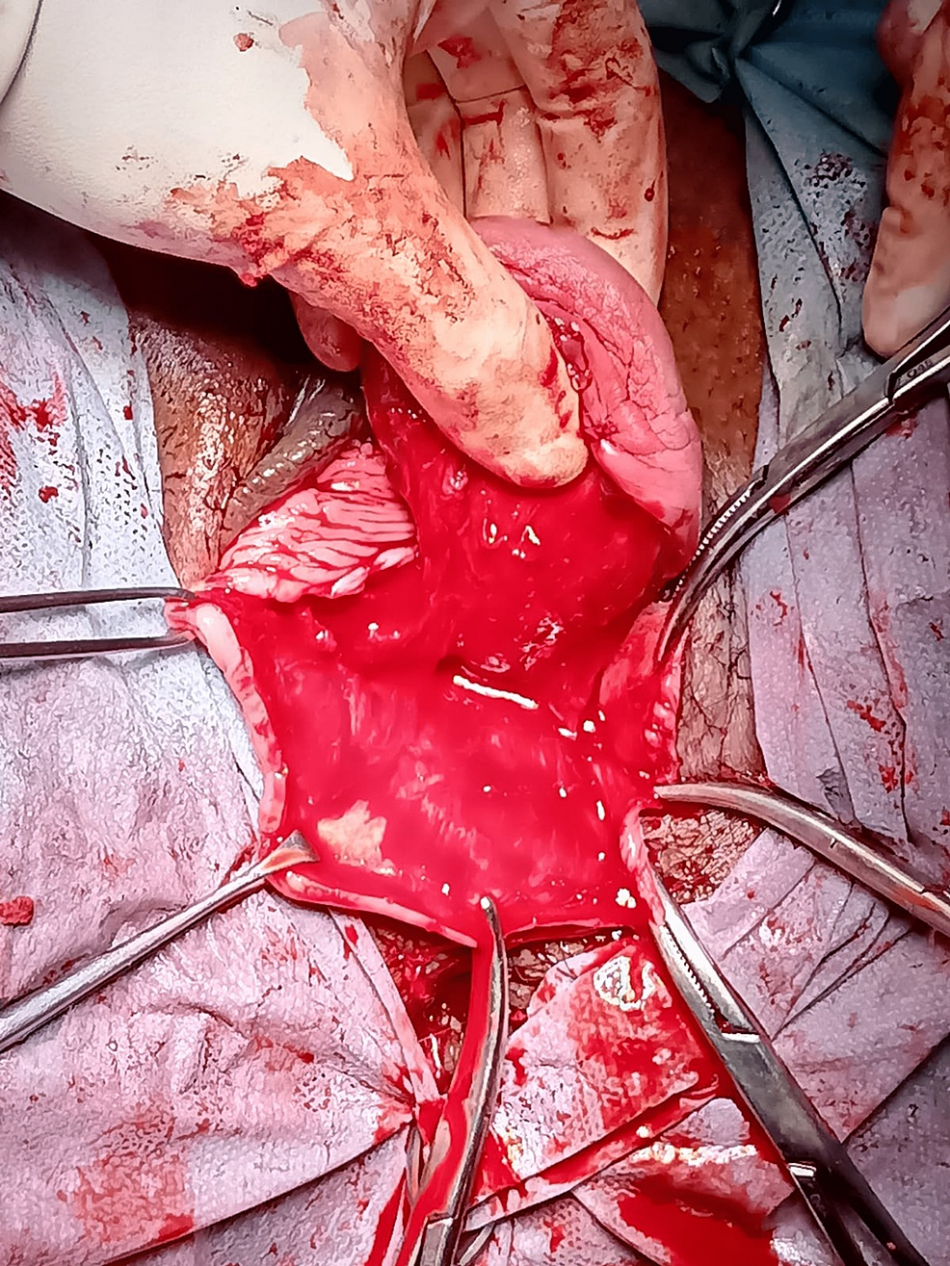
Posterior vaginal flap separated.

**Figure 5:**
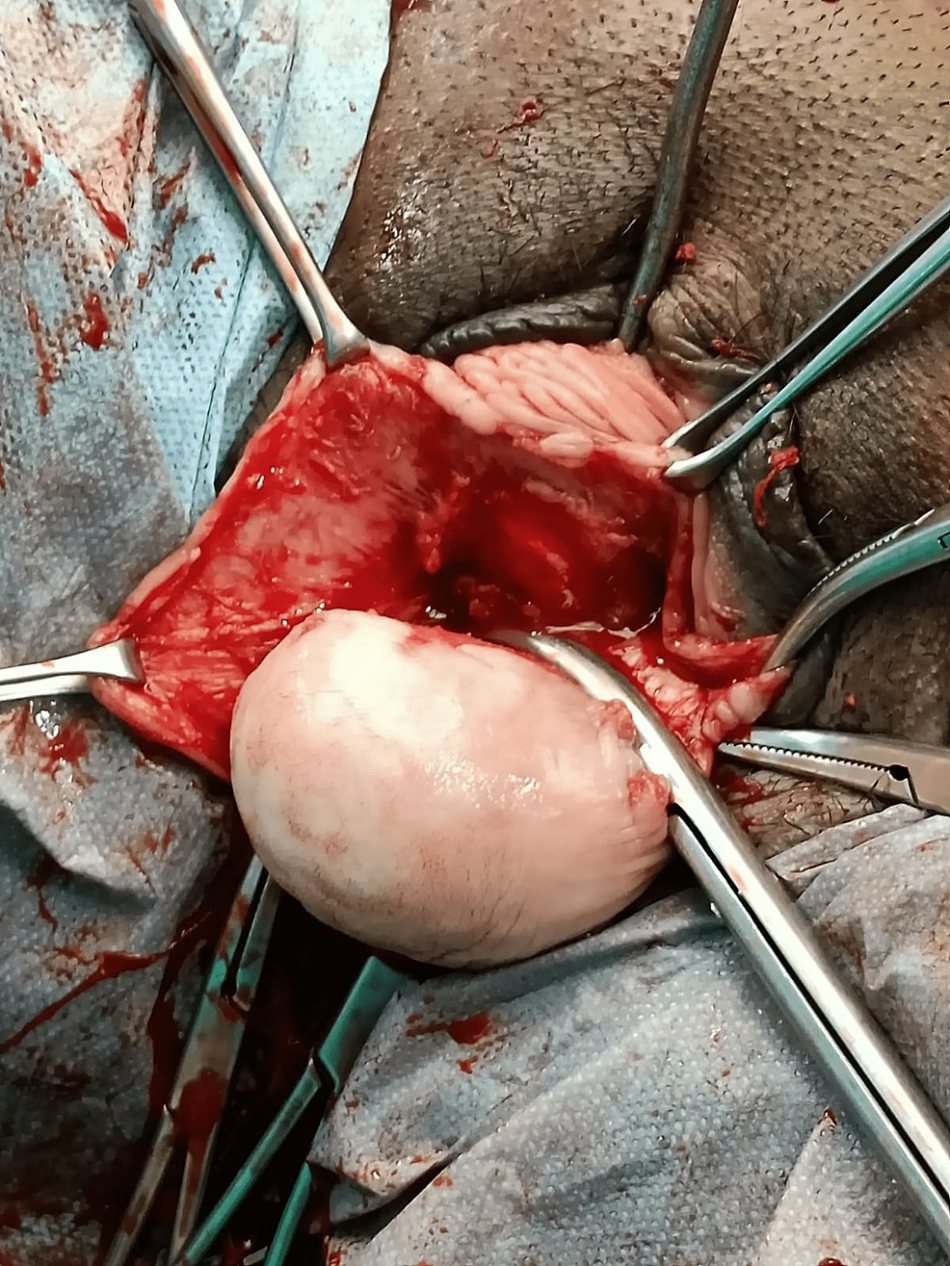
Cyst wall freed from the vagina, and pedicle arising from the left anterolateral wall clamped.

Under spinal anesthesia, the patient was positioned in dorsal lithotomy. A diagnostic urethral cystoscopy was conducted to rule out the urethral diverticula. Using a metal catheter inside the urethra, the plane of dissection between the urethra and cyst wall was identified. A curvilinear incision over the anterior vaginal wall was made by the vaginal route, and the cyst separated from the vaginal wall in toto without rupture. Complete cyst excision was performed uneventfully (Figures 2-5).

**Figure 6:**
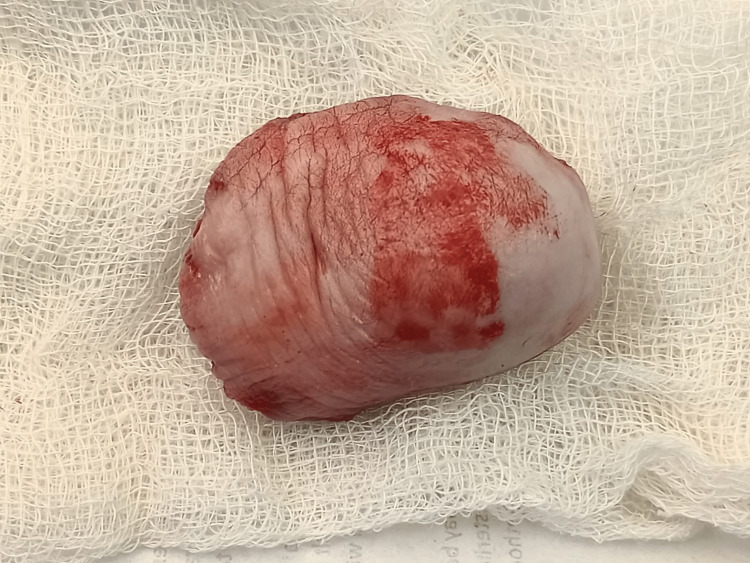
The cyst was removed intact.

**Figure 7:**
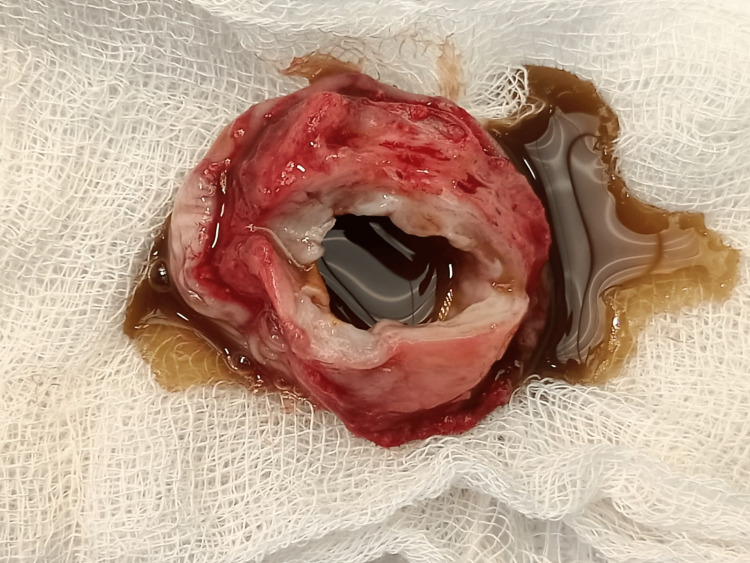
A cut section of the cyst showing thick mucinous content.

The cyst wall was thick and filled with mucinous content on the cut section (Figures 6, 7).

**Figure 8:**
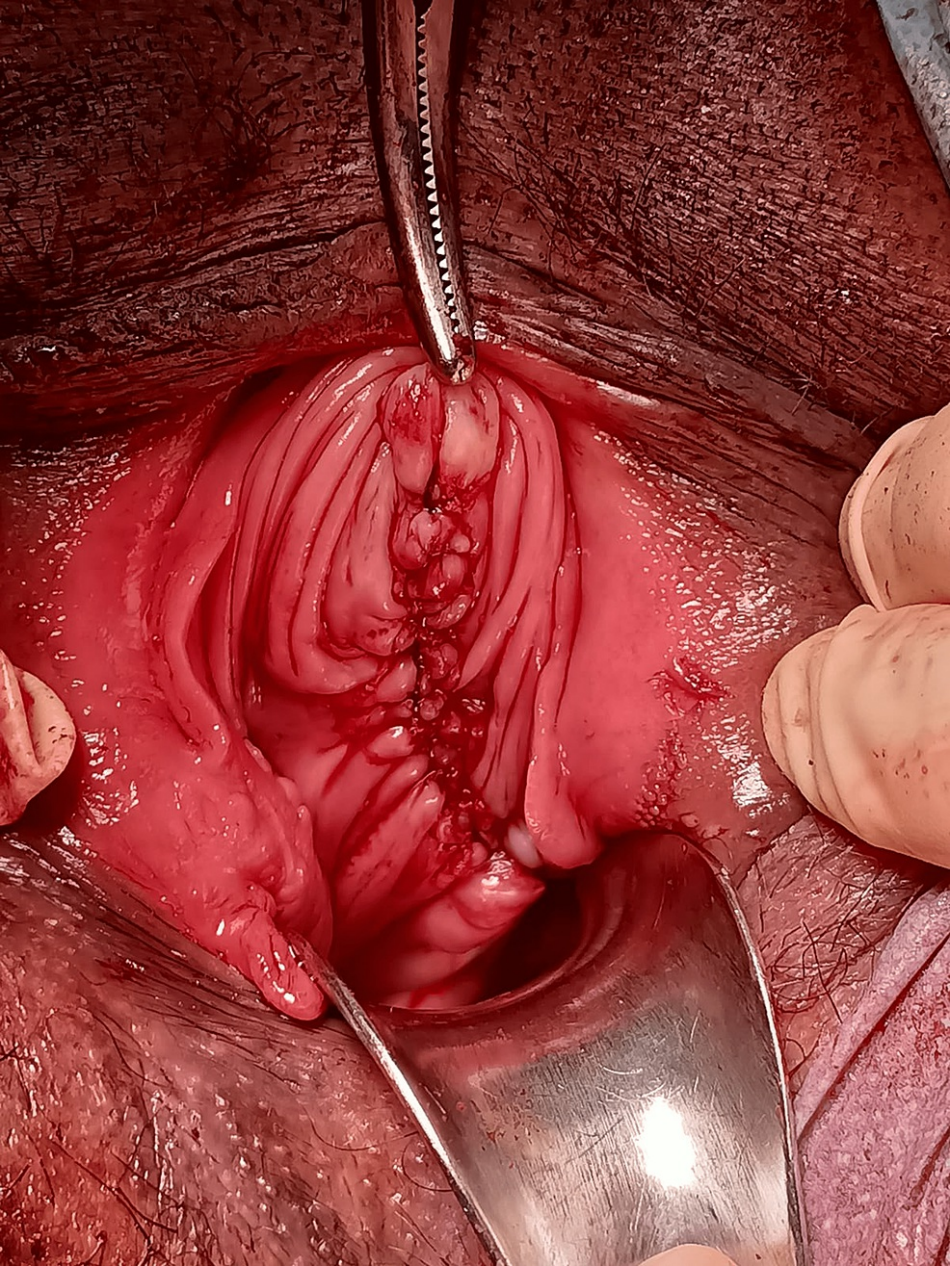
Extra vaginal flap cut and sutured.

**Figure 9:**
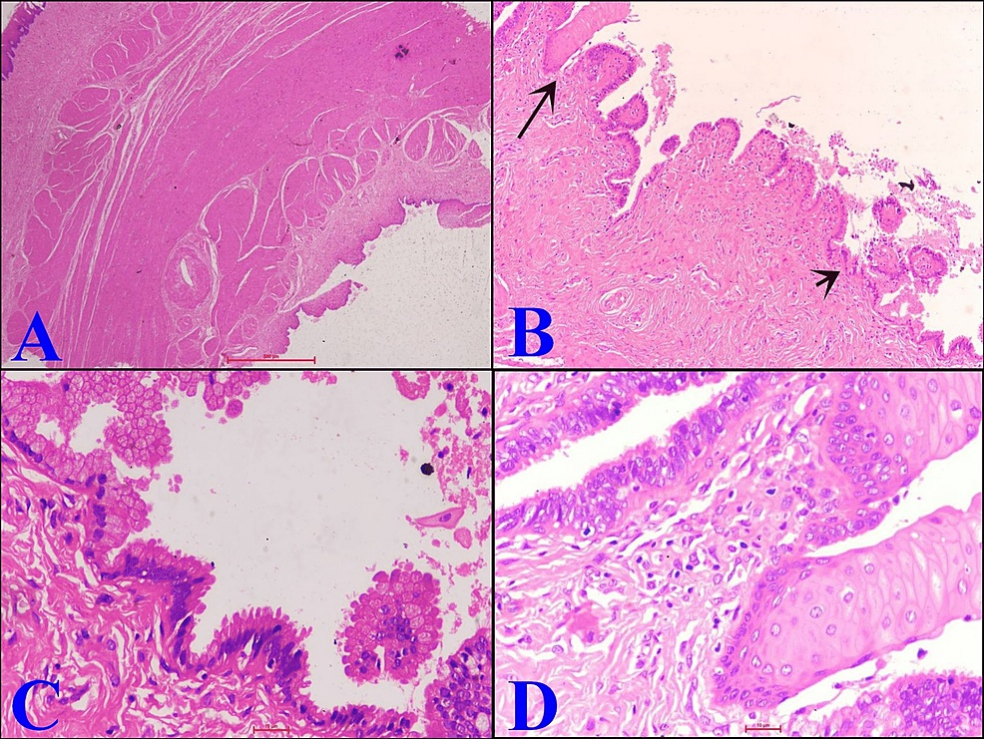
Histopathology images. (A) Skin-covered fibromuscular tissue with the presence of a cyst (H&E; 2×). (B) Cyst wall lined by mucin-secreting columnar cells (short arrow) and focal squamous metaplasia (long arrow) (H&E; 10×). (C) Cyst wall showing both ciliated (tubal type) and mucin-containing columnar epithelium (H&E; 40×). (D) Cyst wall showing focal squamous metaplasia with the presence of mild chronic inflammatory infiltrate in the subepithelium (H&E; 40×). H&E = hematoxylin and eosin

After removing the extra vaginal flap, dead space was obliterated, and vaginal edges were sutured with continuous locking suture using vicryl 0 (Figure 8). Vaginal packing was done, which was found soaked after eight hours of surgery. For this, she was re-evaluated, and a few more hemostatic sutures were made. In the postoperative period, her hemoglobin level was 7.5 mg/dL, for which one unit of packed red blood cells was transfused. Intravenous (IV) antibiotics and analgesics were administered, and she was discharged on postoperative day five. Histopathology report showing mucin-secreting epithelial cells confirmed the cyst as a Mullerian cyst (Figure 9).

**Figure 10:**
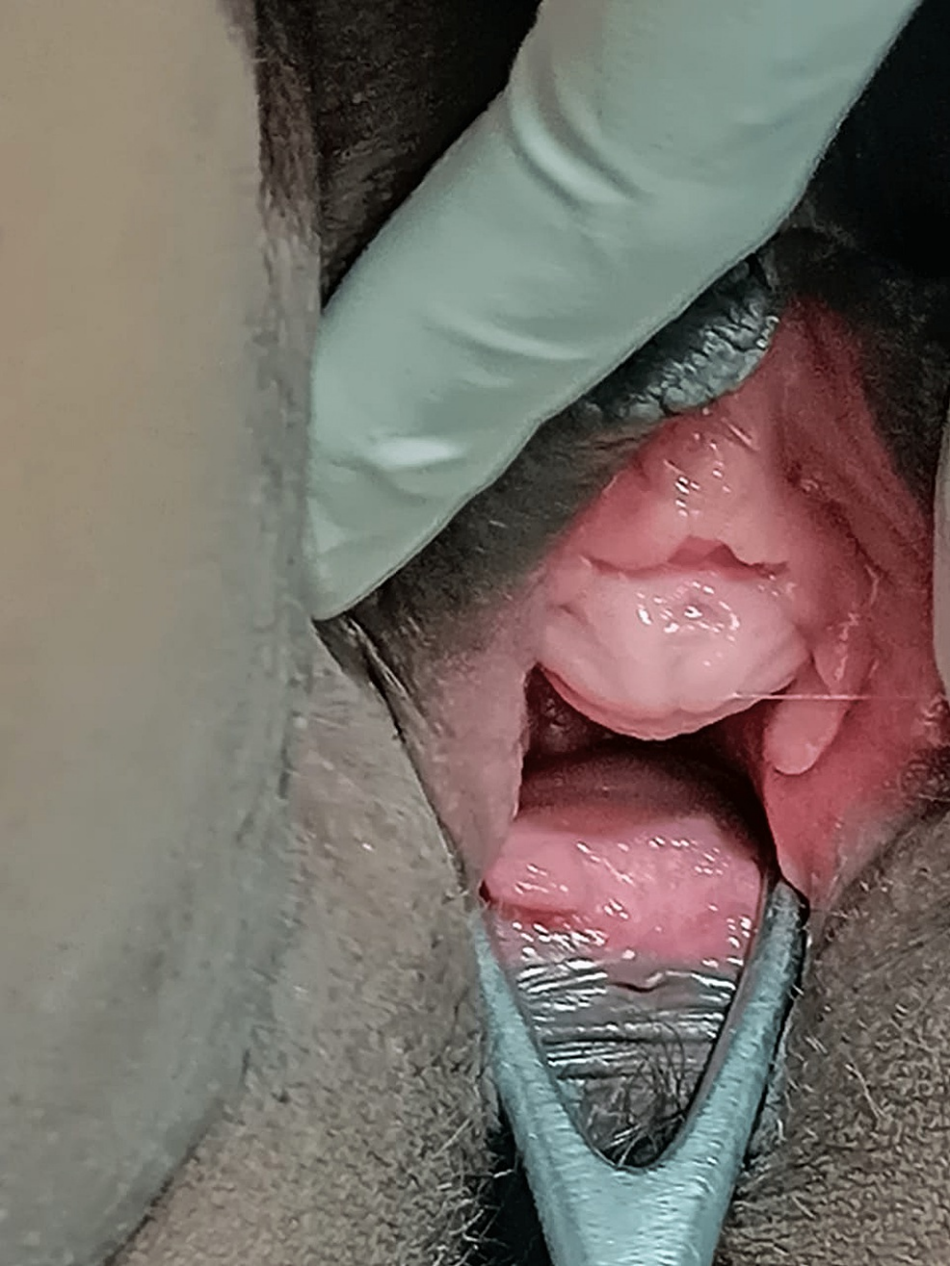
The scar healed at follow-up after three months.

The patient was followed up twice in the outpatient department after two weeks and then after three months. At the three-month follow-up, all stitches were absorbed, wound healing was complete, and the patient was relieved of symptoms (Figure 10).

## Discussion

In a female fetus, the ovary passes down from the lumber region to the pelvic region along with the mesentery having both the Mullerian duct and the Wolffian duct. The mesentery itself later forms the mesosalpinx, Mullerian ducts fuse to form a uterine tube, while the Wolffian ducts may persist as a remnant in the form of an ill-defined cord running in the mesosalpinx parallel to the uterine tube, known as the Gartner’s duct [6]. The different possible types of vaginal cysts are Mullerian cysts, Gartner’s cysts, Bartholin cysts, epidermal inclusion cysts, urethral diverticula, and endometriotic cysts. Of these, Mullerian cysts are the most common (40%) type and are almost always benign as malignant transformation to adenocarcinoma has been reported only once in the literature [6]. Mullerian cysts can form anywhere in the vagina, unlike Gartner’s cysts which always form along the lateral wall of the vagina [5]. Gartner’s cysts account for 12% of all vaginal cysts [7]. They usually remain <2 cm in size, with the peak incidence in the fourth decade [2]. In about 25% of cases, they persist till adulthood, and in about 1% of cases, lead to cyst formation [8]. The most common site is the right anterolateral wall of the vagina [9]. Urethral diverticula may be congenital or acquired and diagnosed with cystoscopy and imaging [10]. Endometriotic cysts appear as bluish or brownish nodules in the posterior fornix and are associated with dysmenorrhea [4]. Bartholin cysts form due to blockage of its duct and are in the posterolateral lower third of the vagina, on the inner side of the labia majora at the junction of its anterior two-thirds and posterior one-third [5]. Large Bartholin cysts may also cause dyspareunia. Epidermal inclusion cysts are usually found on the posterior vagina at the lower end, forming due to tags of mucosa which remain embedded into the episiotomy scar or following trauma [1].

Before embarking on the diagnosis of a vaginal cyst, more common entities presenting similarly such as pelvic organ prolapse, cystocele, and urethral diverticula must be ruled out. Pelvic organ prolapse is easily identifiable on clinical examination as the cervix will be the leading part. Cystocele will present with a positive cough impulse, diffuse margins, and transverse rugosities on vaginal mucosa and will be reducible [5]. All these findings are in contrast to vaginal cysts where there will be no cough impulse, margins will be well defined, vaginal mucosa becomes tense with loss of rugosities, and it will be non-reducible. Urethral diverticula present with a triad of postvoid dribbling, dysuria, and dyspareunia. MRI, voiding cystourethrogram, and cystourethroscopy are different diagnostic tools to identify this.

Different vaginal cysts can be differentiated based on their location, clinical examination, and imaging findings. Ultrasonography is the first imaging modality to see the cyst site, size, and association with the neighboring structures. Overall MRI imaging is the imaging choice for vaginal cysts as it gives an excellent view of the vagina and surrounding structures [11]. However, a confirmatory diagnosis is histopathology.

Smaller asymptomatic cysts (<4 cm) require no treatment and can be monitored with follow-up [5]. Larger cysts (>4 cm) are usually symptomatic and need surgical treatment; however, any size of cyst which causes significant problems should be addressed. Different surgical methods are cyst excision, cyst aspiration, marsupialization, unroofing, and puncture. Complete cyst excision is the most commonly performed procedure and alleviates recurrence. However, there is a paucity of literature regarding the progression rate of small cysts and the recurrence rate after different surgical treatments as well.

## Conclusions

A vaginal cyst presents with various symptoms. However, proper history taking, clinical examination, and imaging findings help to find the size and the site, delineate its upper extent, and find any association with other surrounding structures, which will lead to proper surgical planning. Our case was an unusual case of a large vaginal cyst having bladder and bowel symptoms, located in the left anterolateral vaginal wall, and confined to the vagina with no intraperitoneal extension and distinct from other neighboring structures. Complete cyst excision was done. Histopathology was the gold standard and confirmed the cyst as one of Mullerian origin.

## References

[1] Tsiapakidou S, Theodoulidis I, Grimbizis G, Mikos T (2022). Surgical excision of vaginal cysts presenting as pelvic organ prolapse: a case series. Pan Afr Med J.

[2] Kondi-Pafiti A, Grapsa D, Papakonstantinou K, Kairi-Vassilatou E, Xasiakos D (2008). Vaginal cysts: a common pathologic entity revisited. Clin Exp Obstet Gynecol.

[3] Deppisch LM (1975). Cysts of the vagina: classification and clinical correlations. Obstet Gynecol.

[4] Rivlin ME, Meeks GR, Ghafar MA, Lewin JR (2013). Intracystic hemorrhage in a non-endometriotic mullerian vaginal cyst. World J Clin Cases.

[5] Töz E, Sancı M, Cumurcu S, Özcan A (2015). Müllerian cyst of the vagina masquerading as a cystocele. Case Rep Obstet Gynecol.

[6] Lee KS, Park KH, Lee S, Kim JY, Seo SS (2005). Adenocarcinoma arising in a vaginal müllerian cyst: a case report. Gynecol Oncol.

[7] Thapa BD, Regmi MC (2020). Gartner's duct cyst of the vagina: a case report. JNMA J Nepal Med Assoc.

[8] Chandrasekhar V, Rao JV (2017). A case of Gartner’s cyst of the vagina. J Anesth Crit Care Open Access.

[9] Rios SS, Pereira LC, Santos CB, Chen AC, Chen JR, de Fátima B Vogt M (2016). Conservative treatment and follow-up of vaginal Gartner's duct cysts: a case series. J Med Case Rep.

[10] (2022). Urethral diverticulum in women: symptoms, causes and treatment. https://njurology.com/urethral-diverticulum-in-women-symptoms-causes-and-treatment/.

[11] Inocêncio G, Azevedo S, Braga A, Carinhas MJ (2013). Large Gartner cyst. BMJ Case Rep.

